# Supplementary material for: Anion-Dominated Copper Salicyaldimine Complexes—Structures, Coordination Mode of Nitrate and Decolorization Properties toward Acid Orange 7 Dye
Source: Polymers (Basel). 2020 Aug 24;12(9):1910. doi: 10.3390/polym12091910 (PMC7563566; doi:10.3390/polym12091910)
Supplement: Supplementary file 1 [file polymers-12-01910-s001.pdf]

Supporting Information

Anion-Dominated Copper Salicyaldimine Complexes: Structures, Coordination Mode of Nitrate, and Decolorization Properties toward Acid Orange 7 Dye

Meng-Jung Tsai, Chi-Jou Tsai, Ken Lin, and Jing-Yun Wu*

Department of Applied Chemistry, National Chi Nan University, Nantou 545, Taiwan

* Corresponding author.

E-mail address: jyunwu@ncnu.edu.tw (J.-Y. Wu).

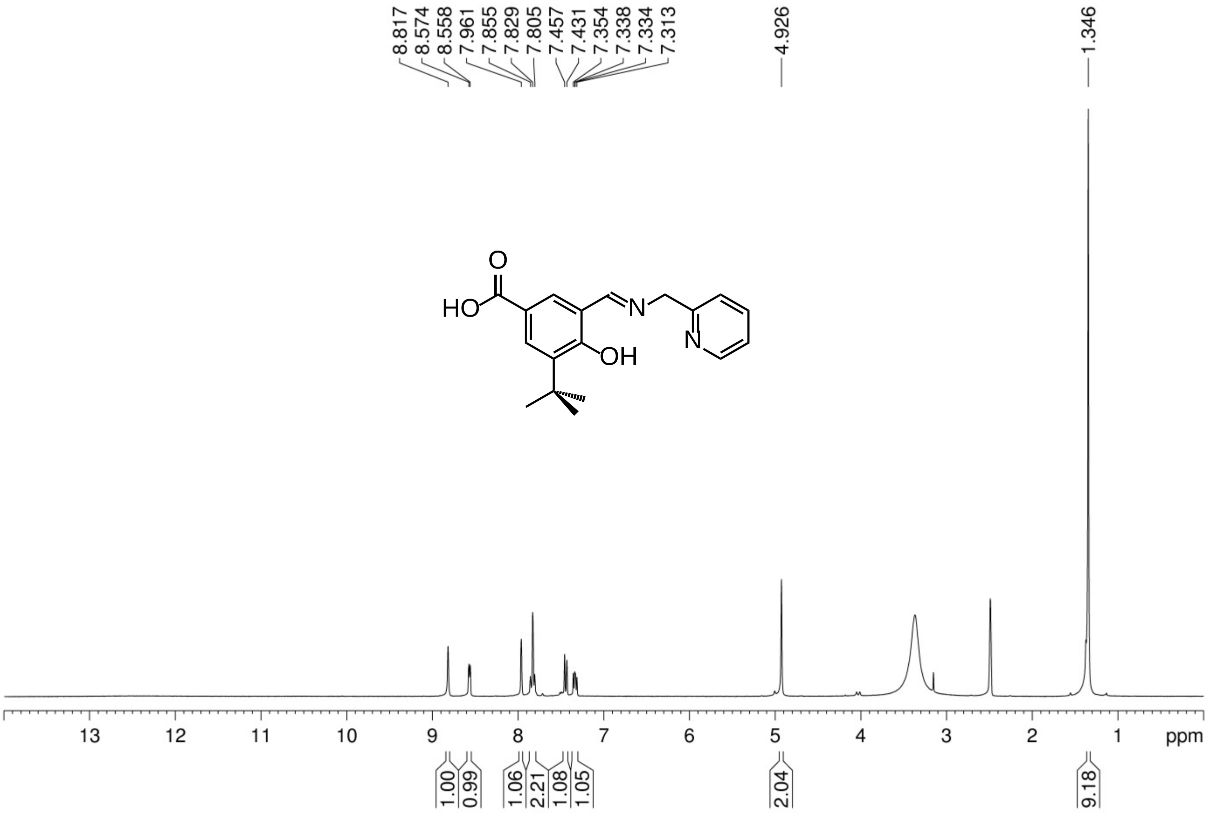

Figure S1. ^{1}H NMR spectrum of $H_2L_{salpyca}$ in DMSO-d_6 at room temperature.

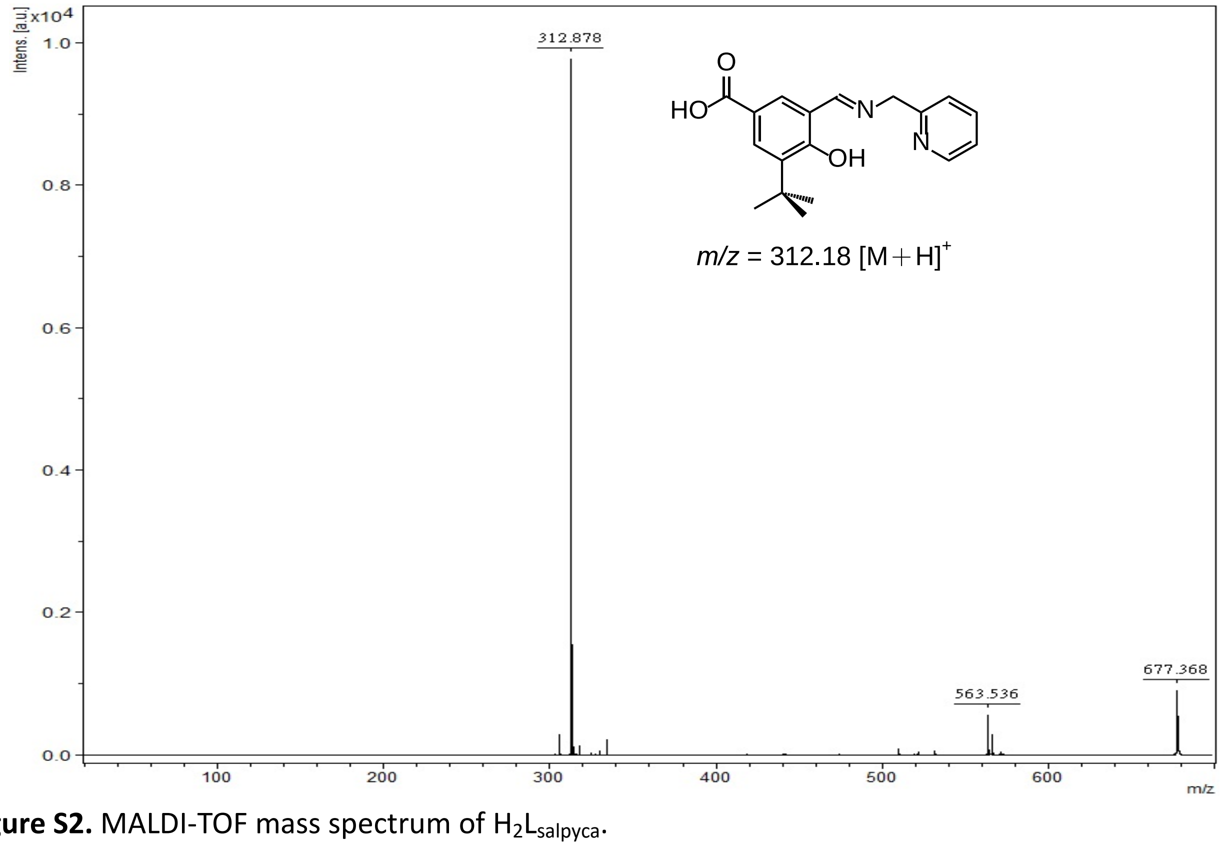

Figure S2. MALDI-TOF mass spectrum of $H_2L_{salpyca}$.

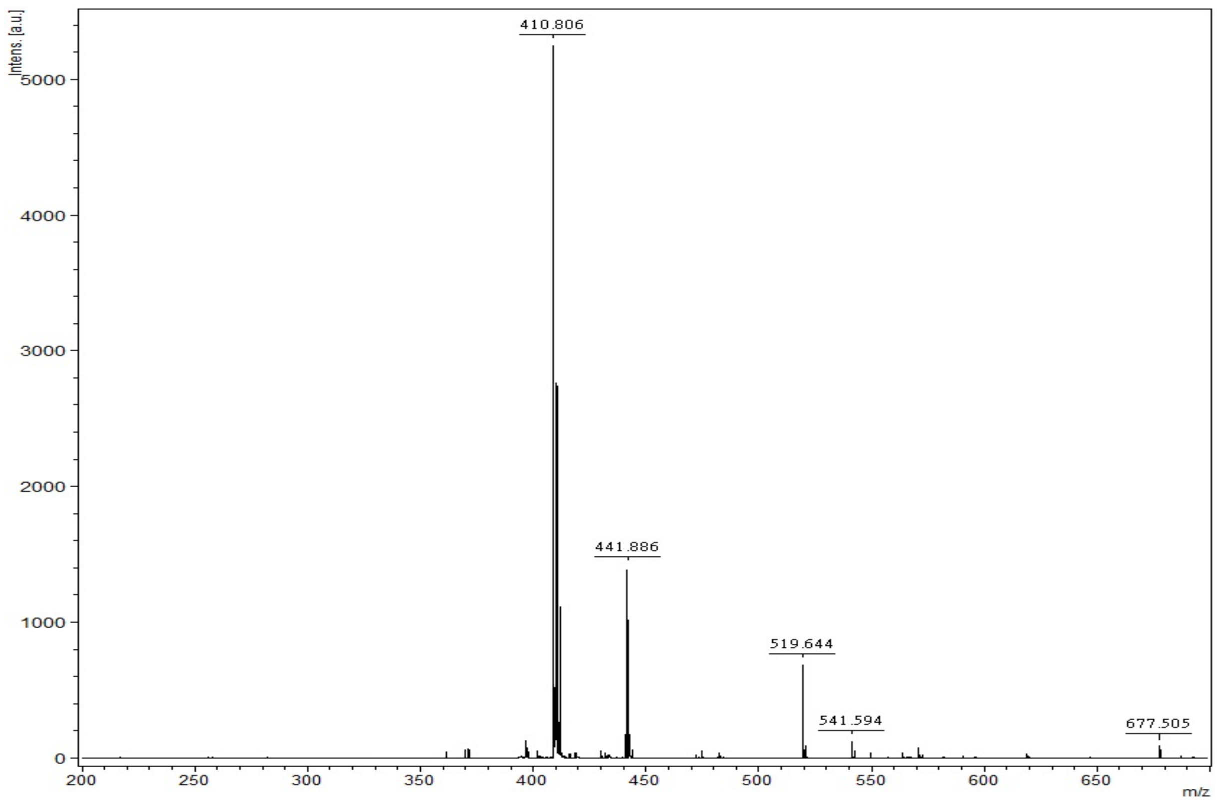

Figure S3. MALDI-TOF mass spectrum of [Cu($HL_{salpyca}$)Cl] (**1**).

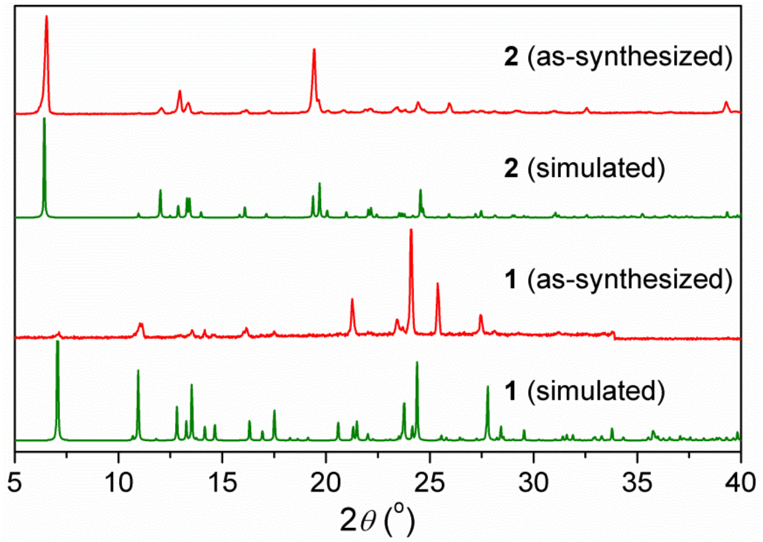

Figure S4. XRPD patterns of **1** and **2**.

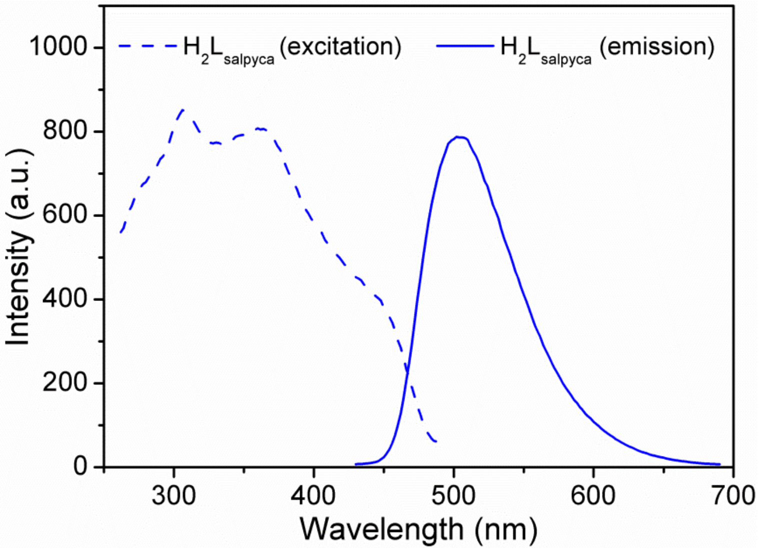

Figure S5. Solid-state excitation and emission spectra of $H_2L_{salpyca}$ (λ_{em} = 505 nm, λ_{ex} = 360 nm).

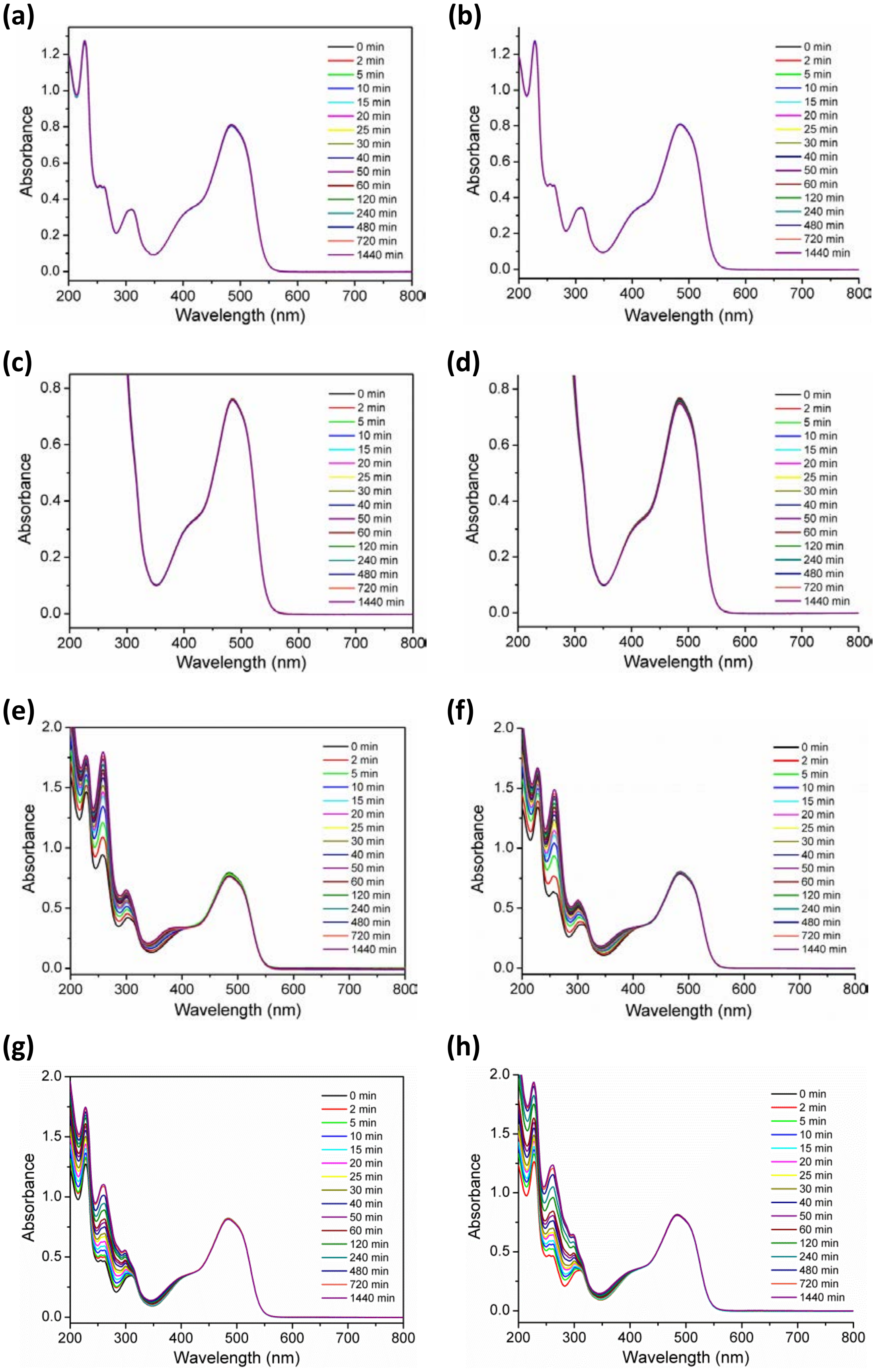

(a)
(b)
(c)
(d)
(e)
(f)
(g)
(h)
Absorbance
Wavelength (nm)
0 min
2 min
5 min
10 min
15 min
20 min
25 min
30 min
40 min
50 min
60 min
120 min
240 min
480 min
720 min
1440 min

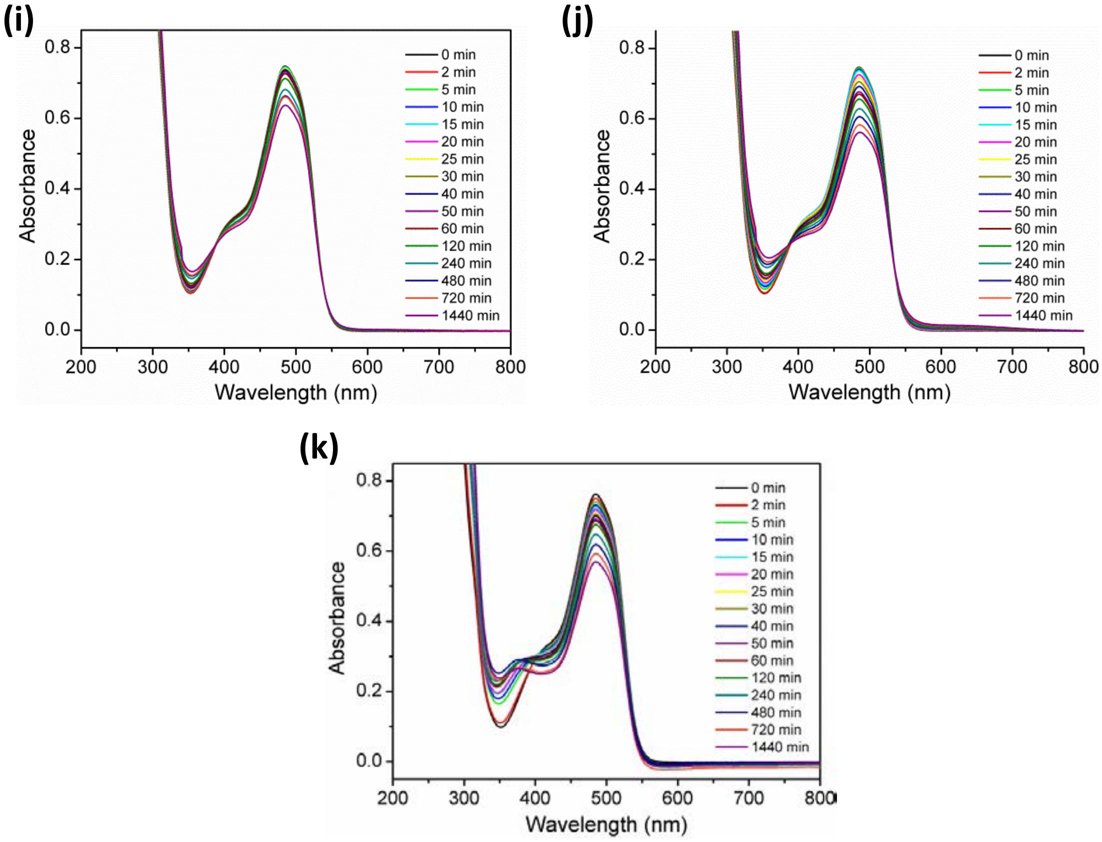

Figure S6. Time-dependent UV/vis spectra of AO7 aqueous solutions under various degradation conditions: (a) AO7 only in dark; (b) AO7 only under daylight, (c) AO7+H_2O_2 in dark; (d) AO7+H_2O_2 under daylight; (e) AO7+**1** in dark; (f) AO7+**1** under daylight; (g) AO7+**2** in dark; (h) AO7+**2** under daylight; (i) AO7+**1**+H_2O_2 in dark; (j) AO7+**1**+H_2O_2 under daylight; (k) AO7+**2**+H_2O_2 in dark.

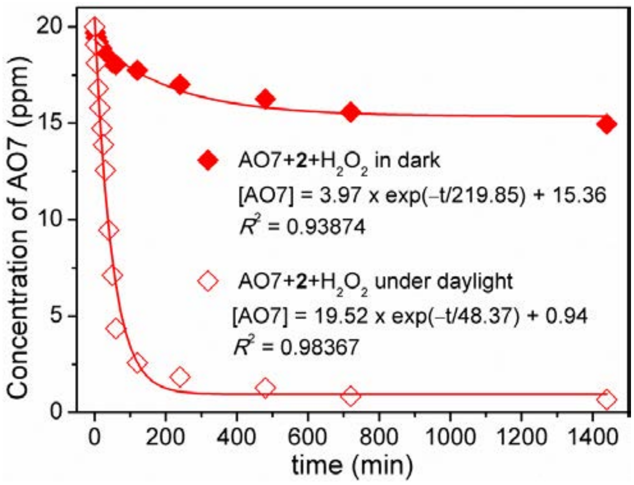

Figure S7. Concentrations of AO7 after degradation by **2**/H_2O_2 versus degradation time in dark conditions and under daylight. Solid lines show the first-order exponential decay.

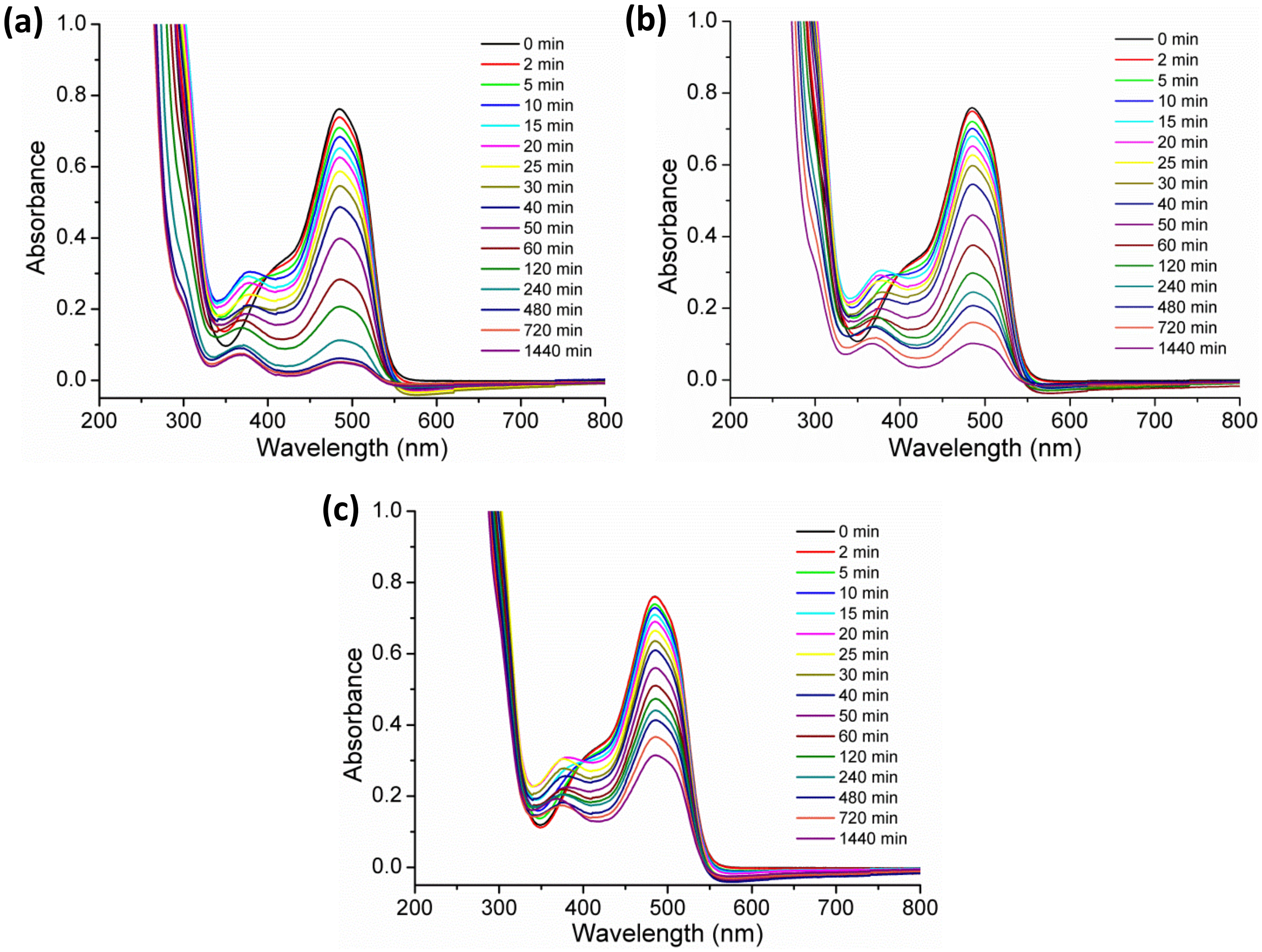

Figure S8. Time-dependent UV/vis spectra of AO7 aqueous solution after the photocatalytic degradation by **2**/H_2O_2 under daylight in different concentrations of **2**: (a) 1 mg **2**/6 mL $AO7_{(aq)}$; (b) 1 mg **2**/15 mL $AO7_{(aq)}$; (c) 1 mg **2**/30 mL $AO7_{(aq)}$.

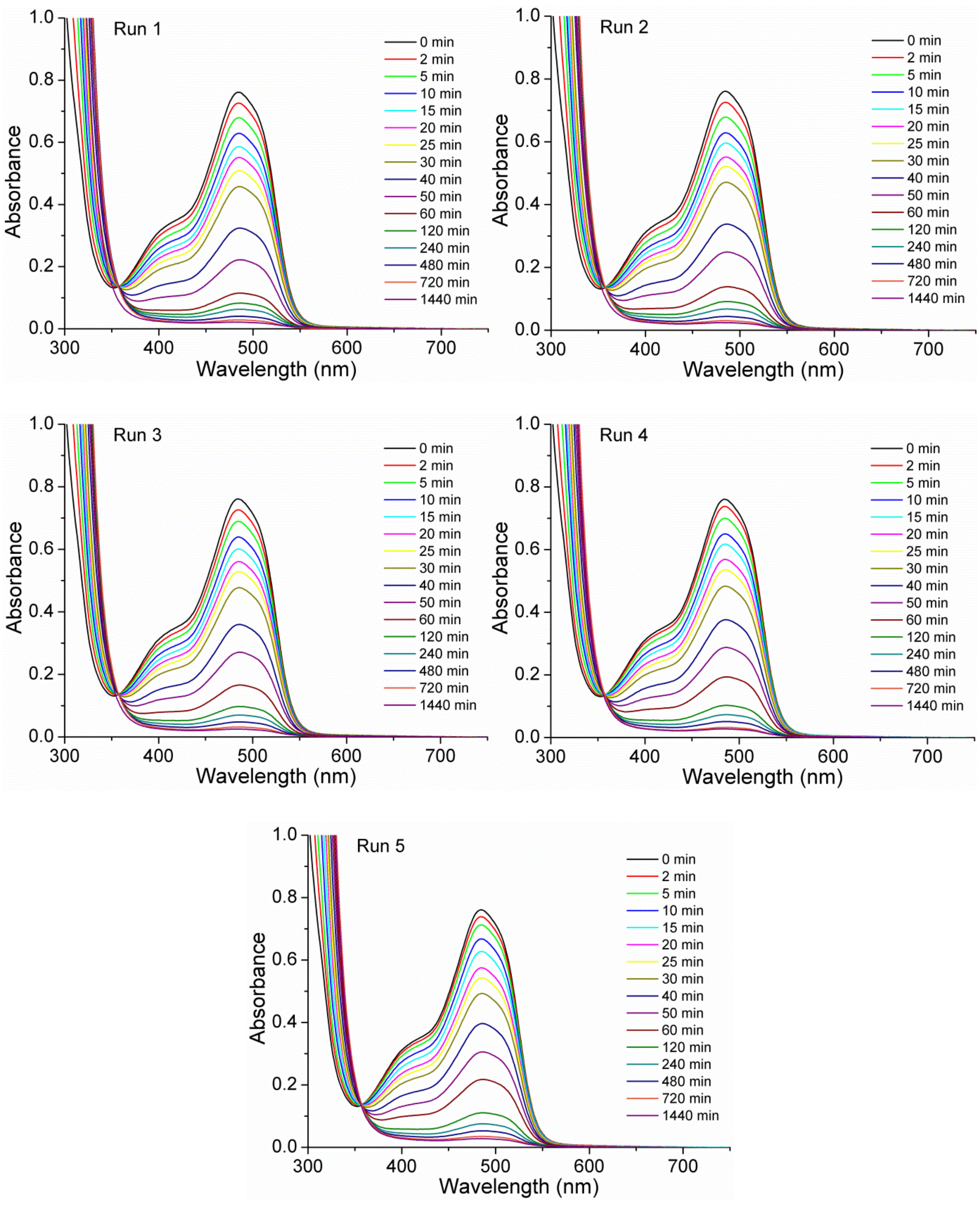

Figure S9. Time-dependent UV/vis spectra of AO7 aqueous solutions for the recycling experiments with the simultaneous existence of **2** and H_2O_2 under daylight.
